# Factors influencing the outcome of image-guided percutaneous drainage of intra-abdominal abscess after gastrointestinal surgery

**DOI:** 10.1007/s00595-013-0504-x

**Published:** 2013-02-14

**Authors:** Yoshiki Okita, Yasuhiko Mohri, Minako Kobayashi, Toshimitsu Araki, Koji Tanaka, Yasuhiro Inoue, Keiichi Uchida, Koichiro Yamakado, Kan Takeda, Masato Kusunoki

**Affiliations:** 1 Departments of Gastrointestinal and Pediatric Surgery, Mie University Graduate School of Medicine, 2-174 Edobashi, Tsu, Mie 514-8507 Japan; 2 Department of Radiology, Mie University Graduate School of Medicine, 2-174 Edobashi, Tsu, Mie 514-8507 Japan

**Keywords:** Postoperative intra-abdominal abscess, Percutaneous abscess drainage, CT-guided drainage

## Abstract

**Purpose:**

To improve the selection of patients for percutaneous abscess drainage (PAD) to treat postoperative intra-abdominal abscess after gastrointestinal surgery, we investigated the factors predictive of outcome.

**Methods:**

Of 143 consecutive patients with symptomatic postoperative intra-abdominal abscess after a gastrointestinal tract resection, 104 who underwent image-guided PAD as the initial treatment were reviewed. We assessed the possible associations between successful PAD and patient-, abscess-, surgical-, and drainage-related variables, and investigated the success rates of PAD for patients with vs. those without the factors related to successful outcome.

**Results:**

Based on monitoring for 1 year after PAD, the success rate of this procedure was 85.6 % (89/104). Multivariate analysis revealed that the interval between surgery and the onset of abscess (*p* = 0.0234) and a single abscess (*p* = 0.0038) were independently associated with a successful outcome. Single late-onset abscess resolved completely within 10 weeks in 91.4 % of these patients.

**Conclusions:**

Despite new strategies aimed at preventing surgical site infection, PAD remains an important factor in the postoperative management of gastrointestinal surgery in Japan. Initial recognition of the day of onset and the number of abscesses are important prognostic factors.

## Introduction

Intra-abdominal abscess is a frequent cause of morbidity and mortality following surgery of the alimentary tract [1, 2]. In the past three decades, advances in image-guided percutaneous abscess drainage (PAD) have provided a safe and effective alternative to surgical drainage [3–7]. Despite the lack of randomized studies comparing percutaneous to surgical drainage, PAD has become a widely accepted treatment for accessible postoperative intra-abdominal abscess, especially in Western countries [8–12]. However, the concepts of treatment for postoperative intra-abdominal abscess after gastrointestinal surgery differ between Japan and Western countries [13]. In Japan, routine abdominal drains are generally placed to facilitate the diagnosis of anastomotic leakage and reduce the risk of intra-abdominal abscess formation [14, 15], although increasing evidence suggests that prophylactic drains do not reduce the incidence of postoperative complications following a variety of intra-abdominal procedures [16]. Routine abdominal drains also play a therapeutic role when intra-abdominal abscess develops after surgery [13]. However, with the increasing use of CT-guided drainage, the indications for PAD have expanded.

The current study focuses on how patient-, surgery-, abscess- and drainage-related factors affect the outcome of PAD, since the implementation of standard surgical site infection prevention policies in Japan. We investigated the effectiveness and safety of PAD, and identified the factors predictive of its successful outcome, to improve the selection of patients who would benefit from this procedure for postoperative intra-abdominal abscess following gastrointestinal surgery.

## Methods

### Patients

Our surgical site infection database identified 143 patients with a symptomatic postoperative intra-abdominal abscess diagnosed after gastrointestinal surgery, between January 2002 and March 2010, at Mie University Hospital. Among these, 104 patients received image-guided PAD as the initial treatment. The 39 patients who did not receive image-guided PAD initially were treated with open surgical drainage (*n* = 16), antibiotic therapy alone (*n* = 16), or transanal drainage (*n* = 7). The study group included 71 men and 33 women, with a mean age of 51 ± 2 years (mean ± SE, range 14–91). The primary diseases and initial surgical procedures are summarized in Tables 1 and 2, respectively. Gastrointestinal surgery was performed for malignant disease in 47 (45.2 %) patients and for inflammatory bowel disease in 41 (39.4 %) patients. Asymptomatic radiographical enteric fistulae without abscess were not included. Intra-abdominal abscess was suspected with the development of such symptoms as abdominal pain, pyrexia, leucocytosis, and shock. The abscess was diagnosed by CT scan in all cases and defined as an infected fluid collection identified by image-guided needle aspiration during image-guided PAD. Recurrent abscess after restorative surgery for previous postoperative intra-abdominal abscess was excluded, so that patients were not included more than once. When severe diffuse peritonitis or septic shock was suspected, open surgical drainage was performed. Patients with an abscess that could not be treated by PAD, in the absence of signs of peritonitis, were treated with antibiotic therapy alone. Transanal drainage was performed for enteric fistula just above the anus, caused by anastomotic leakage after surgery such as low anterior resection, and when PAD carried a risk of injury to other abdominal organs or major vessels.

**Table 1:** Underlying primary disease

	*n*
Colorectal cancer	28
Ulcerative colitis	24
Gastric cancer	19
Crohn’s disease	17
Colon diverticula	4
Acute appendicitis	4
Adhesive small bowel obstruction	3
Others	5

**Table 2:** Initial surgical procedures

	*n*
Gastric surgery	
Total gastrectomy	10
Distal gastrectomy	8
Small intestinal surgery	
Small bowel resection	11
Ileostomy closure	11
Stoma construction	6
Ileoanal reanastomosis	3
Appendectomy	4
Colorectal surgery	
Rectal resection	13
Right hemicolectomy	9
Restorative proctocolectomy with ileal pouch anal anastomosis	9
Subtotal colectomy with ileorectal anastomosis	4
Left hemicolectomy	4
Subtotal colectomy without ileorectal anastomosis	3
Transverse colon resection	3
Sigmoidectomy	2
Ileocolorectal or colorectal anastomosis after Hartman’s procedure	2
Abdominal perineal resection	1
Colostomy closure	1

### Indications and procedure for PAD to treat postoperative intra-abdominal abscess

PAD was attempted as the initial procedure only if an abscess could be accessed without risk of injury to other abdominal organs, if severe diffuse peritonitis was not suspected, and in the absence of septic shock at presentation, based on judgment of the surgeon and interventional radiologist. All procedures were performed under local anesthesia and image guidance: as CT-guided PAD in 83 patients and as ultrasound-guided PAD in 21 patients. The attending interventional radiologist decided on the size and number of catheters used, based on the nature of the fluid obtained at needle aspiration and the extent of the abscess. The catheter size ranged from 8 to 12 F and Pigtail drainage catheters (Skater Drainage Catheter; Angiotech, Stenlose, Denmark) were placed in the abscess cavity using the Trocar method or Seldinger technique. When abscess drainage was insufficient, the catheter was replaced by a thicker one, inserted using an over-the-guidewire technique or it was moved to a position that allowed sufficient drainage. Bags were attached for gravity drainage after placing a stopcock at the external end of the catheter for routine irrigation. Abscess cavities of all patients who underwent PAD were irrigated with natural saline from drainage tubes about 1 week after PAD. No concomitant antibiotics were given before puncture of the PAD and/or during PAD when the abscesses were localized with mild symptoms, based on the judgment of the surgeon. However, concomitant antibiotics were administered to patients with severe symptoms. Antibiotics were initially chosen empirically and changed, if necessary, based on culture and sensitivity results.

### Definition of outcomes

Patients were divided into two groups depending on whether the PAD outcome was successful. Success was defined as complete resolution of the intra-abdominal abscess or enteric fistula after one or more PAD procedures without the need for surgery. Complete resolution of the intra-abdominal abscess was defined as radiological disappearance of the abscess cavity and clinical disappearance of the symptoms. The catheter was removed after CT or fluoroscopy confirmed complete resolution of the fluid collection or enteric fistula. When recurrent intra-abdominal abscesses had been drained and resolved completely, the outcome of PAD was defined as successful. The “success group” did not include any patients in whom PAD was subsequently deemed to have failed in the follow-up period. Failure was defined as the need for elective interval surgery or emergency surgery after PAD. Patients with a postoperative intra-abdominal abscess were monitored for at least 1 year after PAD and their outcomes were judged according to the definitions of success and failure.

### Definition of variables

#### The potential success factors were as follows

*Patient*-*related factors* age at surgery, gender, malignant disease, inflammatory bowel disease, steroid treatment, diabetes mellitus, and laboratory data just before PAD (white blood cell count, hemoglobin, CRP, ALB, choline esterase).

*Surgery*-*related factors* surgical procedure, stoma construction, anastomotic operation, surgical duration, operative blood loss, and wound class.

*Abscess*-*related factors* interval between surgery and onset, interval between onset and PAD, size and number of abscess/es.

*Drainage*-*related factors* drainage procedure, concomitant use of antibiotic therapy, duration of antibiotic therapy, multiple drains, and need for additional PAD.

The surgical procedure and wound class were categorized according to the National Nosocomial Infections Surveillance System. Surgical procedures were divided into gastric surgery (GAST), small bowel surgery (SB), appendectomy (APPY), and colorectal surgery (COLO). The wound class comprised four criteria: clean, clean-contaminated, contaminated, or dirty [17]. The day of onset was defined as the day when patients complained of symptoms related to the abscess. Common presenting symptoms included pyrexia, abdominal tenderness, and abdominal fullness.

Abscess location on CT scans was categorized into nine areas: right subphrenic, Subhepatic/Morson’s pouch, right gutter, left subphrenic/perisplenic, left gutter, peripancreas/lesser sac, pelvis/perirectal, below the abdominal wall, and other interperitoneal. A single abscess was defined as an abscess found in a single location, whereas multiple abscesses were defined as abscesses located in more than two locations. Duration in the cumulative success rate of PAD was defined as the interval between PAD puncture and complete resolution of the abscess.

### Statistical analysis

Quantitative data are expressed as mean ± SE (range). Comparisons between the success group and the failure group were analyzed by the Chi-square test with Yate’s correction and the Mann–Whitney *U* test for quantitative and qualitative variables, using Statview 4.5 software (Abacus Concepts, Berkeley, CA, USA). Univariate analysis was used to examine the relationship between the success of PAD and the variables studied. All variables associated with the failure group resulting in *p* < 0.1 on univariate analysis were examined consecutively by multivariate analysis logistic regression. A *p* value of <0.05 was considered significant. Correlation between the enteric fistulae and a single or late-onset abscess was analyzed by the Chi-square test with Yate’s correction.

## Results

### Outcome after PAD for postoperative intra-abdominal abscess

The success rate of PAD at 1 year was 85.6 % (*n* = 89), although 24 of these patients required repeat drainage. The failure group consisted of six patients who underwent emergency operations for peritonitis, and nine patients who underwent or needed to undergo elective operations for enteric fistulae. Table 3 summarizes the clinical characteristics and outcomes of these 15 patients. Six patients required emergency conversion to open surgical drainage and stoma construction after PAD because of peritonitis originating from enteric fistulae. In eight patients, the enteric fistulae were not closed and elective surgery was needed later. All enteric fistulae during PAD were confirmed by fluoroscopy. In one patient, the enteric fistula persisted for over 1 year and elective surgery was scheduled. All of the ‘failure group’ patients had enteric fistulae. No patient died after PAD in this series. There was one major complication related to PAD; namely, massive bleeding from long-term placement, in a patient from the success group.

**Table 3:** Clinical characteristics and outcome of the 15 patients in the failure group

Case	Age	Gender	Types of primary disease	Procedure of restorative operation	Emergency/Elective	Intervals between initial PAD and restorative operation
1	19	M	UC	Open surgical drainage and stoma construction	Emergency	1
2	22	M	UC	Open surgical drainage and stoma construction	Emergency	1
3	25	F	UC	Open surgical drainage and stoma construction	Emergency	1
4	75	F	Colorectal cancer	Open surgical drainage and stoma construction	Emergency	2
5	91	M	Adhesive small bowel obstruction	Open surgical drainage and stoma construction	Emergency	2
6	79	M	Colorectal cancer	Open surgical drainage and stoma construction	Emergency	5
7	64	M	Colorectal cancer	Reanastomosis without stoma construction	Elective	32
8	30	M	CD	Stoma construction	Elective	57
9	32	M	CD	Reanastomosis with dysfunctional stoma construction	Elective	74
10	38	M	Gastric cancer	gastro-jejunal bypass	Elective	88
11	34	M	CD	Stoma construction	Elective	148
12	25	M	UC	Stoma construction	Elective	171
13	15	F	CD	Reanastomosis without stoma construction	Elective	364
14	37	F	CD	Reanastomosis without stoma construction	Elective	720
15^a^	71	F	Colorectal cancer	–	–	–

*UC* ulcerative colitis, *CD* Crohn’s disease, *PAD* percutaneous abscess drainage

^a^Patients with persistent enteric fistulae for over 1 year

### Factors associated with successful outcome

Univariate analysis showed that a higher white blood cell count tended to be associated with successful PAD, but there were no other differences in patient- and surgery-related factors between the success and failure groups (Table 4). A longer interval between surgery and onset and having a single abscess were also associated with success, but there were no other differences in abscess- and drainage-related factors between the success and failure groups (Table 5). Multivariate analysis was performed using three variables (Table 6): white blood cell count, interval between surgery and onset, and single abscess. Multivariate analysis showed that a longer interval between surgery and onset (odds ratio = 1.248; 95 % CI 1.031–1.510; *p* = 0.0232) and having a single abscess (odds ratio = 7.690; 95 % CI 1.899–31.136; *p* = 0.0042) were significantly associated with the successful outcome of PAD.

**Table 4:** Patient-related and surgery-related factors divided into success and failure groups

Factors	Success group (*n* = 89)	Failure group (*n* = 15)	*p* value
Age at surgery (years)	52 ± 2	44 ± 6	0.2116
Gender (M/F)	61/28	10/5	>0.9999
Malignant disease (Y/N)	44/45	6/9	0.6910
Inflammatory bowel disease (Y/N)	32/57	9/6	0.1396
Preoperative intra-abdominal abscess(Y/N)	7/82	3/12	0.1403
Steroid treatment (Y/N)	22/67	4/11	>0.9999
Diabetes mellitus (Y/N)	8/88	0/15	0.5987
White blood cell count (/mm^3^)	12400 ± 500	11800 ± 2400	0.0663
Hemoglobin (g/dl)	10.0 ± 0.2	10.4 ± 0.5	0.6206
CRP (mg/d)	12.6 ± 0.8	15.0 ± 2.3	0.3548
ALB (g/dl)	3.0 ± 0.1	2.9 ± 0.1	0.3222
Choline esterase (ΔpH)	0.48 ± 0.02	0.50 ± 0.06	0.8464
Categories of surgical procedure (GAST/SB/APPY/COLO)	17/25/4/43	1/7/0/7	0.3521
Stoma construction (Y/N)	34/55	2/13	0.1142
Anastomotic operation (Y/N)	73/16	12/3	>0.9999
Surgical duration (min)	272 ± 13	227 ± 27	0.1612
Operative blood loss (g)	523 ± 53	360 ± 110	0.3920
Wound class (CC/CO/D)	66/13/10	10/4/1	0.4738

*GAST* gastric surgery, *SB* small bowel surgery, *APPY* appendectomy, *COLO* colorectal surgery, *CC* clean-contaminated operation, *CO* contaminated operation, *D* dirty/infected operation

**Table 5:** Abscess- and drainage-related factors divided into “success” and “failure” groups

Factors	Success group (*n* = 89)	Failure group (*n* = 15)	*p* value
Interval between surgery and onset (days)	10 ± 1	6 ± 1	0.0003
Interval between onset and PAD (days)	3 ± 0	4 ± 2	0.1679
Size of abscess (<5 cm)	23/66	2/13	0.4701
Single abscess (Y/N)	63/26	3/12	0.0005
Drainage procedure (CT/US)	73/16	10/5	0.9434
Concomitant use of antibiotics therapy (Y/N)	69/16	4/2	0.3064
Duration of antibiotics therapy (days)	8 ± 1	7 ± 2	0.9268
Multiple drain (Y/N)	24/65	6/9	0.4699
Number of drainage tube	1.5 ± 0.1	1.6 ± 0.2	0.4762
Additional PAD (Y/N)	19/70	5/10	0.4915

*PAD* percutaneous abscess drainage

**Table 6:** Logistic regression analysis for factors associated with successful outcome

	Odds ratio	95 % CI	*p* value
White blood cell count (/mm^3^)	1.038	0.916–1.175	0.5605
Interval between surgery and onset (days)	1.248	1.031–1.510	0.0232
Single abscess (Y/N)	7.690	1.899–31.136	0.0042

### Success rates of PAD for patients with vs. those without factors related to outcome

**Fig. 1:**
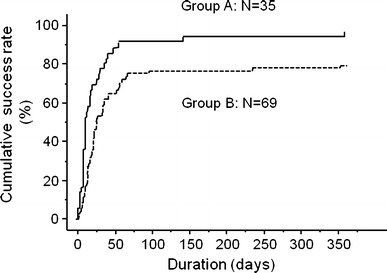
The cumulative success rate of percutaneous abscess drainage in 104 patients increased with therapeutic duration. The success rate was 85.6 % (*n* = 89) after 1 year of follow-up

The median interval between surgery and the onset of intra-abdominal abscess was 8 days, the onset being early (<8 days) in 55 patients and late (>9 days) in 49 patients. Patients were divided into two groups according to the presence or absence of factors related to a successful outcome. Group A (*n* = 35) comprised patients with a single and late-onset abscesses and group B (*n* = 69) comprised patients with multiple and/or early onset abscesses. Figure 1 shows the cumulative success rates of PAD in Groups A and B. The success rate of PAD in Group A was 97.1 % (34/35), with a median PAD period of 14 days and complete resolution within 10 weeks (70 days) in 91.4 % (32/35) and sometime after 10 weeks in 5.7 % (2/35). The success rate of PAD in Group B was 79.7 % (55/69), with a median PAD period of 21 days and complete resolution within 10 weeks in 76.8 % (53/69) and sometime after 10 weeks in 2.9 % (2/69).

### Association between the absence of enteric fistulae and a single and late-onset abscess

All enteric fistulae were confirmed during PAD by fluoroscopy and detected in 53 patients (51.0 %). No significant correlation was found between the absence of enteric fistulae and a single and late-onset abscesses (*p* = 0.1660).

### Success rate of PAD for patients with abscess related to an enteric fistula

The success rate of PAD among patients with an abscess related to enteric fistulae was 71.7 % with no difference in outcome between small intestinal fistulae and large intestinal fistulae (*p* = 0.8036). Factor XIII concentrate was injected during drainage for five patients with an enteric fistula, resulting in success in four. The success rate of PAD was 92.9 % (13/14) for patients with a single and late-onset abscess related to enteric fistulae but only 64.1 % (25/39) for patients with multiple and/or early onset-abscesses related to enteric fistulae.

## Discussion

PAD was first described in the late 1970s and in 1981; Gerzof et al. [3] reported a success rate of 86 % when used to treat intra-abdominal abscesses in 67 patients. Subsequently, it was demonstrated that the effectiveness and safety of PAD [8, 18–20], which over the last 30 years, made the transition from a revolutionary to a routine procedure, replacing open surgical drainage, except in the most difficult or inaccessible cases. Using univariate analysis, several authors have identified the factors predictive of the failure of PAD, including enteric fistulae, multiple or loculated abscesses, large abscesses, necrotic tissue, and pancreatic localization [3, 8, 11, 21–24].

Percutaneous drainage has been used in the management of complex abscesses, including multiple abscesses, those associated with fistulae, splenic abscesses, and infected fluid collections whose drainage route traversed normal organs) [7]. The use of PAD for patients with complex abscesses has been suggested to offer significant therapeutic benefits, even though it may not be curative and surgery could still be required [4, 25]. A study of the literature revealed a success rate of 45–88 % for PAD treating complex abscesses [20, 26–28].

Some postoperative intra-abdominal abscesses associated with anastomotic leaks lead to diffuse peritonitis or abnormal communications between the gastrointestinal tract and the skin, with or without persistent clinical sepsis [29]. Approximately, one-third of enterocutaneous fistulae will close spontaneously with proper supportive care, control of sepsis, and nutritional support [30]. Wainstein et al. [31] reported that fistulae healed spontaneously in 46 % of patients, within a mean period of 90 days (range 8–370 days). Peng et al. [32] reported that irrigation-suction through the drainage tubes was effective in approximately 75 % of patients with leakage, without the need for surgical intervention. In their study, the median irrigation time when leakage occurred was 21 days (range 5–55 days). In the current study, the median interval between PAD puncture and complete resolution was 19 days (range 2–357 days). There are wide variations in the PAD period for postoperative intra-abdominal abscesses. The vast majority of studies on PAD for postoperative intra-abdominal abscesses have reported technical success based on short-term results, without long-term follow-up of individual patients, even though some had enteric communications [33–35]. However, long-term follow-up is necessary to accurately assess the complete resolution rate for postoperative intra-abdominal abscesses after PAD.

It has been reported that postoperative abscesses are significantly more likely than non-postoperative abscesses to be improved by PAD [36]. However, no published studies, except for that of Benoist et al., have analyzed patients who underwent PAD as the initial therapy for postoperative intra-abdominal abscesses, to find the factors predictive for success using multivariate regression analysis [33, 34, 37]. Benoist et al. examined the factors predictive of PAD failure for postoperative intra-abdominal abscesses in 73 patients and found that the absence of antibiotic therapy and an abscess diameter of <5 cm were the only two independent factors associated with failure of PAD. These authors also showed that even complex postoperative abscesses, such as those associated with enteric fistulae, were not associated with failure. The overall success rate in the current study was 85.6 % after 1 year of follow-up, which is consistent with the high success rates reported in previous studies [20, 26, 27, 37]. Multivariate analysis showed that a shorter interval between surgery and onset, and having multiple abscesses, but not the use of antibiotic therapy or the size of the abscess, were related to failure of PAD. Benoist et al. reported that patients with small abscesses in the failure group required repeat surgery for persistent or recurrent sepsis after drain removal, probably because of incomplete drainage. In our study, when abscess drainage was insufficient, the catheter was exchanged for a thicker one or it was moved to a position that allowed sufficient drainage. This adaptable drainage technique may have been an important factor in improving the outcome. Benoist et al. also reported that the absence of antibiotic therapy was an independent factor for failure of PAD. We did not give antibiotic therapy to patients with mild symptoms of an abscess; thus, the indications for antibiotic therapy may have been different in the two studies. No previous study has identified the interval between surgery and abscess onset as a significant predictive variable for failure of PAD. However, the early onset of postoperative intra-abdominal abscesses may reflect their severity.

In this study, the success rate of PAD was 78.0 % (32/41) for patients with inflammatory bowel disease, whereas it was 90.5 % (57/63) for those without inflammatory bowel disease (*p* = 0.1396); however, inflammatory bowel disease was not related to the unsuccessful outcome statistically. Six of nine patients who underwent, or would undergo, elective surgery for an enteric fistula had inflammatory bowel disease. Moreover, the proportion of patients with inflammatory bowel disease needing elective surgery for an enteric fistula was 14.6 % (6/41), whereas the proportion of patients without inflammatory bowel disease needing elective surgery for an enteric fistula was 4.8 % (3/63) (*p* = 0.1636). All in all, the proportion of patients with inflammatory bowel disease, who needed elective surgery for an enteric fistula, was not higher statistically.

We evaluated the rate of complete resolution within the first 10 weeks, and compared the median PAD periods in Groups A and B. The rate of complete resolution of single and late-onset abscesses within the first 10 weeks after PAD was very high. All of the patients with abscesses in the failure group also had enteric fistulae, demonstrating that enteric fistula was related to the unsuccessful outcome of PAD. There was no significant correlation between the absence of enteric fistulae and single and late-onset abscess, so single and late-onset abscesses did not indicate an absence of enteric fistulae. Even if abscesses related to enteric fistula were present, the success rate of PAD for single and late-onset abscesses was very high.

Enteric fistulas during PAD were detected in 51.0 % of the patients in this study; however, the types of enteric fistula that tended to be cured by PAD were not analyzed. Campos et al. [38] and Gonzalez-Pinto et al. [39] reported that spontaneous closure was more likely for low-output fistulas, and those caused by surgery, those with free distal flow, healthy surrounding bowel, simple fistula with no associated abscess cavity, a fistula tract >2 cm, a fistula tract not epithelialized, an enteral defect <1 cm, a low fistula output, and no co-morbidity.

The potential limitations of our study include that it was a retrospective cohort series with a study population that was heterogeneous because of the wide variations in the disease and operative procedures. The decision of whether to employ PAD was at the discretion of the surgeon, which could have resulted in selection biases, and the data would be difficult to extrapolate to general patients undergoing gastrointestinal surgery. Multivariate logistic regression analysis was performed to minimize the effect of confounding factors. In addition, the effectiveness of conservative therapy such as nutritional management, the administration of octreotide and wound care, should be taken into consideration when examining factors that affect enteric fistulae closure. In this study, we focused on the relationship between patient-, surgery-, abscess- and drainage-related factors, and the outcome after PAD with long-term follow-up.

In conclusion, we evaluated the outcome of PAD in patients with intra-abdominal abscesses after recent gastrointestinal surgery. PAD is a safe and effective procedure for postoperative intra-abdominal abscess, with a high success rate and a low complication rate. We found that a single abscess and its late onset are independent predictors for a successful outcome of PAD. Conservative treatment within the first 10 weeks may be a better choice for patients with single and late-onset abscesses, even if persistent enteric fistulae are present. Initial recognition of the day of onset and the number of abscesses is important for providing prognostic information, which may subsequently influence the choice of treatment. Despite the establishment of modern strategies aimed at preventing surgical site infection, PAD remains an important factor in the postoperative management of gastrointestinal surgery in Japan. Further studies applying these prognostic models to different populations and larger numbers of patients are needed to validate and refine the models and generalize the results.
